# Major components of metabolic syndrome and adiponectin levels: a cross-sectional study

**DOI:** 10.1186/1758-5996-6-26

**Published:** 2014-02-26

**Authors:** Anize D von Frankenberg, Filipe V do Nascimento, Lucas Eduardo Gatelli, Bárbara L Nedel, Sheila P Garcia, Carolina SV de Oliveira, Pedro Saddi-Rosa, André F Reis, Luis H Canani, Fernando Gerchman

**Affiliations:** 1 Post-Graduate Endocrinology Program, Faculdade de Medicina, Universidade Federal do Rio Grande do Sul (UFRGS), Porto Alegre, Brazil; 2 Metabolism Unit, Endocrinology Division, Hospital de Clínicas de Porto Alegre (HCPA), Porto Alegre, Brazil; 3 Endocrinology Unit, Universidade Federal de São Paulo (UNIFESP), São Paulo, Brazil; 4 Hospital de Clínicas de Porto Alegre. Rua Ramiro Barcelos, 2350 – Prédio 12. 4° andar, 90035-003 Porto Alegre, RS, Brazil

**Keywords:** Adiponectin, Metabolic syndrome, Obesity

## Abstract

**Background:**

Adiponectin is a major regulator of glucose and lipid homeostasis by its insulin sensitizer properties. Since decreased insulin sensitivity is linked to metabolic syndrome (MS), decreased adiponectin levels may be related to its development. The purpose of the study was to investigate the relationship between adiponectin levels and MS.

**Methods:**

Firstly, we cross-sectionally examined subjects with or without MS submitted to an oral glucose tolerance test at Hospital de Clínicas de Porto Alegre (n = 172). A replication analysis was performed in subjects (n = 422) undergoing cardiac angiography at Hospital São Paulo. Subchronic inflammation (US-CRP), coagulation marker (fibrinogen), insulin sensitivity and resistance (Matsuda ISI and HOMA-IR) were estimated. Plasma total and high molecular weight (HMW) adiponectin were measured.

**Results:**

Total and HMW adiponectin levels were lower in MS subjects (P < 0.05). Total adiponectin levels were lower in the presence of high waist circumference, low HDL-cholesterol and elevated triglyceride criteria in both samples and by elevated blood pressure and glucose criteria in Porto Alegre. HMW adiponectin levels were lower in the presence of low HDL-cholesterol, elevated triglycerides, and glucose criteria. Total adiponectin levels were positively related with HDL-cholesterol and ISI Matsuda, negatively related with waist circumference, glucose, triglycerides, HOMA-IR, and US-CRP and not related with blood pressure. While adjusting for sex and age, increased adiponectin levels remained associated with a reduced prevalence ratio for MS in both cohorts (P = 0.001).

**Conclusions:**

Adiponectin levels decreased with increasing number of MS criteria, and it is in part determined by its relationship with HDL, triglycerides and abdominal adiposity.

## Background

Obesity is a major public-health problem worldwide. It has been associated with the development of metabolic syndrome (MS) [1] which is an interrelated cluster of risk factors for cardiovascular disease (CVD) and type 2 diabetes [2,3] such as hyperglycemia, raised blood pressure, elevated triglyceride levels, low high-density lipoprotein cholesterol levels, and central obesity [4]. Data from the NHANHES study has shown that 22.9% of United States adult population had MS in 2009/10 [5].

The growing rates of this obesity-related syndrome have spurred the search for greater insight about mechanisms contributing to the development of MS, especially those reflecting a dysfunction of adipose tissue, which probably plays a major role in its development [6].

Adiponectin, a hormone expressed in adipose tissue and encoded by the ADIPOQ gene (chromosome 3q27), plays an important role in regulating insulin sensitivity, glucose and lipid metabolism besides anti-inflammatory and anti atherogenic properties [7]. Its high molecular weight (HMW) isoform is the major responsible for these functions [8]. In the presence of obesity, adiponectin release is down regulated resulting in reduced circulating levels [9].

In order to investigate the relationship between total and HMW adiponectin levels and the presence of MS we measured its levels in two different cohorts. We were also interested in finding out which MS components are mostly related to adiponectin levels. Additionally, we analyzed how insulin resistance, sensitivity and subchronic inflammation were related to adiponectin levels.

## Methods

### Subjects

In order to determine the relationship between MS and adiponectin, we undertook a two-stage study using data from two cohorts. In the first stage, the relationship between adiponectin and MS was assessed in consecutive subjects who were referred for ambulatory care in the Metabolism Unit of Hospital de Clínicas de Porto Alegre, Federal University of Rio Grande do Sul (n = 172), whose glycemic status was not previously defined. Exclusion criteria included clinically significant autoimmune diseases, uncompensated hypo or hyperthyroidism, malignant disease that may affect 5-year survival, stage IV or V chronic kidney disease, AIDS, pregnancy/lactation, dementia, cirrhosis, hepatitis, alcohol or illicit drug abuse, glucocorticoid or anti-retroviral treatment, and malnutrition. The protocol was approved by the institutional review board of Hospital de Clínicas de Porto Alegre and the subjects provided written informed consent.

In order to confirm the relationship between adiponectin levels and MS we performed a second stage study (replication analysis) with data from the Endocrinology and Cardiovascular Units of the hospital of the Federal University of São Paulo (São Paulo cohort, UNIFESP) of 422 consecutive subjects who had complete assessment of MS criteria and underwent cardiac angiography for investigation of coronary heart disease, according to an indication of their clinician as previously described [10]. Exclusion criteria included creatinine clearance <50 mL/min, abnormal thyroid function, presence of active inflammatory disease and neoplasia. The protocol was approved by the institutional committee of ethical practice of UNIFESP and subjects provided written informed consent.

### Study procedures and assays

Subjects from the Porto Alegre and the São Paulo cohorts underwent a standard evaluation which included medical history, physical examination and anthropometric measurements. Ethnicity was classified based on self-reported skin color and recorded as white and non white. Current smoking was defined by active consumption in the last three months. Habitual alcohol consumption was accessed by a yes or no question. Subjects were classified as physically active if they practiced moderate-intensity activity for at least 150 minutes per week. Body weight was recorded in light clothing without shoes. Height was measured on a stadiometer. BMI was calculated by weight (kg)/height (m^2^) [11]. Waist circumference was taken at the midpoint between the lower costal margin and the iliac crest measured to the nearest 0.5 cm.

In the Porto Alegre cohort, blood pressure measurements were performed one week after withdrawal of all antihypertensive medications whereas in the São Paulo cohort subjects were not withdrawal of medications in use. Office blood pressure were measured by the arm blood pressure oscillometric monitor device OMRON® (H-003D) with cuff adjusted for arm circumference while the participant was seated, in the right arm three times by auscultation, and the mean of the last two measurements was used to estimate systolic and diastolic arterial blood pressure in both cohorts. For a better understanding of the relationship between adiponectin and arterial blood pressure, ambulatory 24-h blood pressure monitoring (ABPM) was performed by oscillometry (Spacelabs 90207), with a 15-min interval during the daytime and a 20-min interval during the nighttime in 129 subjects from the Porto Alegre cohort. Subjects were advised to maintain their usual daily activities. Sleep time was recorded as the period between the time when the patient went to bed and the time when the patient woke up the next morning. The means of 24-h, daytime, and nighttime systolic and diastolic blood pressure were recorded.

Blood samples were drawn after an overnight fast for analysis of lipids (total, HDL cholesterol, and triglycerides), glycated hemoglobin (HbA1c), insulin, and total adiponectin in subjects from both cohorts. HMW adiponectin was measured in blood samples of subjects from the São Paulo cohort. Ultra-sensitive C-reactive protein (us-CRP) and fibrinogen were measured in blood samples of subjects from the Porto Alegre cohort. Subjects from the Porto Alegre cohort were also submitted between 8:00 and 9:00 AM to a 75-g oral glucose tolerance test (OGTT) in which both glucose and insulin were measured at 0, 30, 60, 90 and 120 minutes. All subjects were classified according to glucose tolerance status [12] and presence of MS [4].

### Assays

Serum total and HDL-cholesterol, triglycerides, insulin, HbA1c, US-CRP and fibrinogen were measured in the Clinical Pathology Unit. Cholesterol and triglycerides were determined by means of an enzymatic method (Ádvia 1800); insulin by chemiluminescence (Centaur XP); HbA1c by high performance liquid chromatography (Tosoh Plus); US-CRP by turbidimetry (Ádvia 1800) and fibrinogen by coagulometric method (BCS).

In the Porto Alegre cohort, plasma samples for adiponectin were analyzed in duplicate by using ELISA kits (Invitrogen®; intra-assay coefficient of variation [CV] < 3.84% and inter-assay CV < 5.50%). Plasma adiponectin study samples respectively presented an intra and inter-assay CV of 2.99% and 4.26%. In the São Paulo cohort, total and HMW adiponectin levels were measured in plasma samples using commercial ELISA kits (EZHADP-61 K and EZHMWA-64 K, respectively, Millipore, Saint Charles, MO). Intra- and interassay CV were respectively 7.4% and 10.6% for total adiponectin and 3.41% and 9.0% for HMW adiponectin.

### Classification of metabolic syndrome

MS was defined as the presence of three out of five criteria described below: high waist circumference (≥ 80 cm for women and ≥ 94 cm for men); elevated triglyceride levels (≥ 150 mg/dL [1.7 mmol/L]) or drug treatment for elevated triglycerides levels; reduced HDL-cholesterol (<40 mg/dL [1.0 mmol/L] for men and <50 mg/dL [1.3 mmol/L] for women) or drug treatment for reduced HDL-cholesterol levels; elevated blood pressure (systolic ≥ 130 mm Hg or diastolic ≥ 85 mm Hg) or antihypertensive drug treatment; and elevated glucose (fasting plasma glucose ≥100 mg/dL [6.1 mmol/L]) or drug treatment for hyperglycemia according to Consensus Societies/Joint Interim Statement [4]. Since subjects from the Porto Alegre cohort were submitted to the OGTT, we have also used the 2 h-plasma glucose criteria for impaired glucose tolerance (≥ 140 mg/dL [7.8 mmol/L]) in order to define the glucose criteria [4].

### Classification of glucose tolerance

Based on fasting and 2 h-plasma glucose concentrations, subjects from the Porto Alegre cohort were categorized according to the American Diabetes Association criteria as having normal glucose tolerance (NGT: fasting plasma glucose [FPG] <100 mg/dL [6.1 mmol/L] and 2 h-plasma glucose level <140 mg/dL [7.8 mmol/L]); impaired fasting glucose (IFG; FPG 100-125 mg/dL [6.1–6.9 mmol/L] and 2 h-plasma glucose level <140 mg/dL [7.8 mmol/L]), or impaired glucose tolerance (IGT; FPG <100 mg/dL [6.1 mmol/L] and 2 h-plasma glucose level 140-199 mg/dL [7.8–11.0 mmol/L]) and diabetes (FPG ≥126 mg/dL [7.0 mmol/L] and/or 2-h PG ≥ 200 mg/dL [11.1 mmol/L) [12]. Subjects with IFG and/or IGT were considered to have prediabetes and this was also used to define the glucose criteria of the MS. Subjects from the São Paulo cohort were classified based on fasting glucose as described above and/or using HbA1c levels according to the American Diabetes Association criteria as NGT (FPG <100 mg/dL [6.1 mmol/L] and HbA1c <5.7%); prediabetes (FPG 100-125 mg/dL [6.1–6.9 mmol/L] and/or HbA1c between 5.7 and 6.4%), and diabetes (FPG ≥126 mg/dL [7.0 mmol/L] and/or HbA1c ≥ 6.5%).

### Estimation of insulin sensitivity, insulin resistance, inflammation and endothelium dysfunction

Indices of insulin sensitivity or insulin resistance were determined, including: the homeostatic model assessment of insulin resistance (HOMA IR = [fasting glucose (mmol/L) * fasting insulin (μU/mL)]/22.5) [13] and the Matsuda insulin sensitivity index (Matsuda ISI) that was calculated as 10,000/√ [fasting glucose (mmol/L) × fasting insulin (mU/L)] × [mean glucose × mean insulin during OGTT] [14]. Subchronic inflammation was estimated by US-CRP and coagulation marker by fibrinogen [15,16].

### Statistical analysis

Data were expressed as absolute number (%), mean ± standard deviation (SD) or median (P25-P75). To compare demographic, clinical, and laboratory data utilizing the presence or the different components of MS, the chi-square test, independent-samples t test and one-way ANOVA were used as appropriate. Prevalence of MS by adiponectin tertiles was compared by chi-square test. Variables with a non-normal distribution were log transformed before analyses. The significance of the correlations was examined by using the parametric Pearson's correlation coefficient for normally distributed or log transformed variables. To test the independent association of adiponectin and MS, multivariate regression analyses were performed using three different Poisson regression models. Prevalence ratio and 95% CI for continuous variables are shown for a 1-SD-magnitude increase (equal to 7.063 μg/mL). The first model contained MS as the dependent variable and age, gender and adiponectin as the independent variables. In the second model, US-CRP and HOMA-IR were added. In the third model, HOMA-IR was replaced by ISI-Matsuda into the model. We did not include as independent variables those that define a criterion for MS in order to avoid double correction. A P value or a P for trend < 0.05 was chosen as the level of significance. Calculations were made by using SPSS (version 19.0; SPSS Inc, Chicago).

## Results

### Subjects’ characteristics

#### Porto Alegre cohort

The Porto Alegre cohort comprised 172 subjects, of whom 124 (72%) were females. The clinical and laboratory characteristics are summarized in Table 1. Subjects were subdivided by absence (21%) or presence (79%) of MS. Although these two groups did not differ by gender distribution, ethnicity, smoking status, alcohol consumption, physical activity, total cholesterol, and fibrinogen subjects with MS were older and presented higher HbA1c levels, HOMA-IR, and US-CRP than those without MS. Prediabetes and type 2 diabetes prevalence were higher in the presence of MS. BMI, waist circumference, fasting plasma glucose, triglycerides, and office blood pressure were also greater in subjects with MS. By ambulatory blood pressure monitoring, 24-h, day and night systolic blood pressures were also greater in subjects with than without MS (Table 1).

**Table 1:** Subjects’ demographic, clinical and laboratory characteristics according to the presence of metabolic syndrome (MS)

	**Porto Alegre cohort MS**			**São Paulo cohort MS**		
	**Absence**	**Presence**	**P value**^**a**^	**Absence**	**Presence**	**P value**^**a**^
N	36	136	-	38	382	-
Female sex	28 (78%)	96 (71%)	0.531	10 (26%)	181 (47%)	0.010
Age (years)	48 ± 12	54 ± 11	0.010	59 ± 12	60 ± 10	0.647
Etnicity (% of white)	28 (78%)	114 (84%)	0.676	20 (51%)	233 (61%)	0.286
Tabagism	5 (14%)	18 (13%)	0.740	26 (67%)	302 (79%)	0.334
Habitual alcohol consumption	3 (8%)	11 (8%)	0.937	5 (13%)	10 (3%)	0.016
Physical activity			0.082			0.061
Sedentary	17 (46%)	79 (58%)		18 (46%)	111 (29%)	
Active (≥150 min per week)	19 (54%)	57 (42%)		21 (54%)	271 (71%)	
BMI (Kg/m^2^)	28 ± 6	33 ± 6	-	23 ± 3	29 ± 5	-
Overweight	12 (33%)	48 (36%)	-	9 (24%)	130 (34%)	-
Obesity	12 (33%)	79 (59%)	-	1 (3%)	149 (39%)	-
Waist circumference (cm)						
Females	91.4 ± 14.2	105.5 ± 12.5	-	80.7 ± 8.0	97.8 ± 12.4	-
Males	101.1 ± 16.8	106.7 ± 11.8	-	84.9 ± 6.2	100.6 ± 1.1	-
Glucose Tolerance Status			<0.001			<0.001
Normal Glucose Tolerance	32 (89%)	16 (12%)		20 (53%)	50 (13%)	
Prediabetes	1(3%)	76 (56%)		12 (32%)	123 (31%)	
Type 2 diabetes	3 (8%)	44 (32%)		6 (16%)	209 (56%)	
Fasting plasma glucose (mg/dL)	91 ± 11	114 ± 41	-	100 ± 24	126 ± 47	-
2 h-plasma glucose (mg/dL)	111 ± 43	191 ± 81	-	-	-	-
HbA1c (%)	5.5 ± 0.6	6.4 ± 1.2	0.001	5.7 ± 0.8	6.9 ± 1.6	<0.001
HOMA-IR	1.6 (1.1 – 2.4)	3.3 (1.9 – 4.7)	0.002	0.5 (0.3 – 0.6)	1 (0.6 – 1.6)	<0.001
Total cholesterol (mg/dL)	201 ± 41	205 ± 42	0.749	272 ± 48	270 ± 54	0.861
HDL- cholesterol (mg/dL)	55 ± 13	47 ± 12	-	46 ± 12	38 ± 10	-
Triglycerides (mg/dL)	100 ± 40	162 ± 91	-	96 ± 26	163 ± 92	-
US-CRP (mg/L)	1.8 (1.5 – 4.6)	4.0 (5.4 – 8.3)	0.003	-	-	-
Fibrinogen (mg/dL)	349 (300 – 385)	384 (372 – 412)	0.107	-	-	-
Systolic Blood Pressure (mm Hg)	125 ± 19	144 ± 22	-	138 ± 24	141 ± 23	-
Diastolic Blood Pressure (mm Hg)	79 ± 11	87 ± 13	-	79 ± 15	80 ± 13	-
Systolic 24-h Blood Pressure (mm Hg)	118.4 ± 12.6	134.3 ± 15.2	0.010	-	-	-
Diastolic 24-h Blood Pressure (mm Hg)	71.5 [65 – 79.3]	79.0 [70.5 – 87.0]	0.087	-	-	-
Systolic daytime Blood Pressure (mm Hg)	121.3 ± 12.8	137.9 ± 15.1	0.010	-	-	-
Diastolic daytime Blood Pressure (mm Hg)	75.0 [68.3 – 83.0]	83.0 [75.0 – 90.0]	0.107	-	-	-
Systolic nighttime Blood Pressure (mm Hg)	112.3 ± 13.3	126.6 ± 17.0	0.023	-	-	-
Diastolic nighttime Blood Pressure (mm Hg)	65.6 ± 10.0	71.5 ± 11.9	0.114	-	-	-
Medicines:						
Antihypertensive	8 (22)	68 (52)	0.014	20 (54)	316 (86)	<0.001
Statin	3 (9)	23 (18)	0.671	11 (28)	235 (61)	<0.001
Hypoglycemic	0 (0)	0 (0)	0.998	9 (23)	85 (20)	0.854

Data expressed as absolute number (%), mean ± SD or median (P25-75). SI conversion factors: triglycerides, mg/dL × 0.01129 = mmol/L; cholesterol, HDL-cholesterol, and LDL-cholesterol, mg/dL × 0.02586 = mmol/L; glucose, mg/dL × 0.0556 = mmol/L. ^a^P value for comparisons between two groups was tested by χ^2^ test for categorical variables or Student’s t test for continuous variables and were adjusted for age and sex by multiple logistic regression analysis. Since the sample was grouped by the presence of MS, P values were not expressed for comparison of its components (all P values were < 0.05).

**Figure 1:**
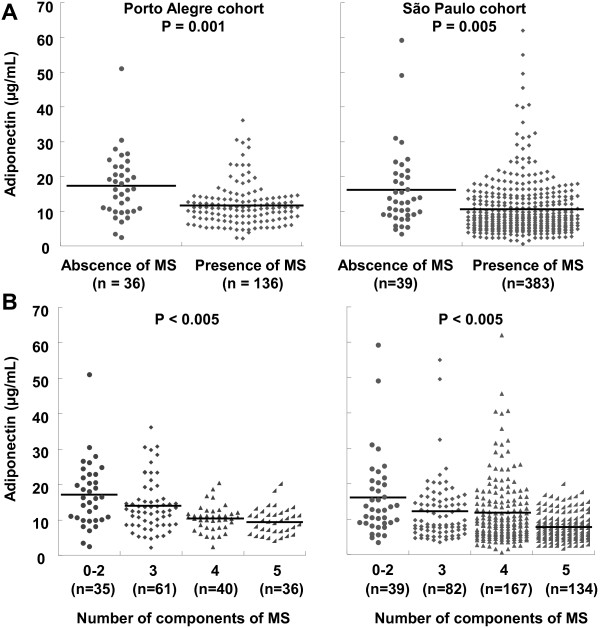
**Adiponectin levels according to the presence of metabolic syndrome (MS).** Comparison by independent T test **(A)**. Adiponectin levels according to the number of components of MS. Comparison by *ANOVA***(B)**.

Plasma adiponectin was lower in the presence of MS (11.0 [7.9 – 13.7 μg/mL] vs 16.4 [10.2 – 22.7 μg/mL]; median [P25-P75], p < 0.001) (Figure 1A) and decreased significantly with an increasing number of MS criteria (Figure 1B). Furthermore, when participants were stratified into three groups by adiponectin tertiles, the prevalence of MS decreased from the lowest to the highest adiponectin tertile (tertile 1 = 89.5%, tertile 2 = 87.9%, and tertile 3 = 59.6%; P for trend < 0.001).

While comparing by each MS criterion, adiponectin levels were significantly lower in the presence of following criteria: high waist circumference (12.4 [8.1 – 14.5 μg/mL] vs 18.5 [11.9 – 26.3 μg/mL]; P = 0.002), low HDL-cholesterol (10.9 [7.3 – 13.2 μg/mL] vs 15.6 [10.0 – 20.4 μg/mL]; P <0.001), elevated triglycerides (10.4 [7.1 – 12.6 μg/mL] vs 14.3 [8.7 – 18.7 μg/mL]; P = 0.001), elevated glucose (11.8 [8.0 – 13.7 μg/mL] vs 15.7 [9.9 – 20.2 μg/mL]; P = 0.032), and elevated blood pressure (12.1 [8.0 – 14.0 μg/mL] vs 15.8 [9.7 – 20.6 μg/mL]; P = 0.030).

**Figure 2:**
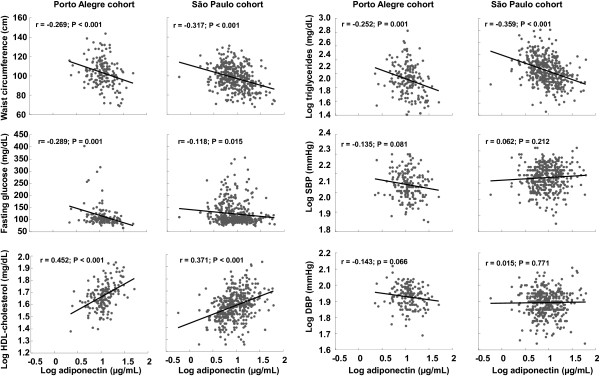
**Relationship between adiponectin levels and metabolic syndrome criteria.** DBP = diastolic blood pressure; SBP = systolic blood pressure.

To examine the relationship between adiponectin levels and the different MS components, we determined their correlations. Adiponectin levels were positively correlated with HDL-cholesterol (r = 0.452, P < 0.001) and negatively correlated with waist circumference (r = -0.269, p < 0.001), fasting glucose (r = -0.289; P = 0.001), and triglycerides (r = -0.252, p < 0.001). There was no significant correlation between adiponectin concentrations and systolic (r = -0.135; P = 0.081) or diastolic (r = -0.143; P = 0.066) office blood pressures (Figure 2). There was also no significant correlation between adiponectin concentrations and systolic 24-h (r = -0.087; P = 0.414), systolic daytime (r = -0.071; P = 0.504), systolic nighttime (r = -0.093; P = 0.383), diastolic 24-h (r = -0.042; P = 0.695), diastolic daytime (r = -0.030; P = 0.779), and diastolic nighttime (r = -0.042; P = 0.692) blood pressures.

Furthermore, adiponectin was negatively related to US-CRP (r = -0.154; P = 0.047) whereas this relationship was not found with fibrinogen (r = -0.048; P = 0.552). Regarding resistance and insulin sensitivity indexes (ISI), adiponectin was negatively associated with HOMA-IR (r = -0.218; P = 0.005) and positively associated with Matsuda ISI (r = 0.191; P = 0.014).

#### São Paulo cohort

In order to confirm the relationship between adiponectin levels and MS we performed a second stage study in 422 subjects who were submitted to cardiac angiography for evaluation of coronary heart disease as indicated by their clinician, of whom 190 (45%) were females (Table 1). Subjects were subdivided by absence (9%) or presence (91%) of MS. Age, ethnicity, smoking status, physical activity, and total cholesterol were similar between groups. However, alcohol consumption was lower in the presence of MS. As we have already observed in the Porto Alegre cohort, glucose tolerance decreased whereas HbA1C and HOMA-IR increased in the presence of MS. BMI, waist circumference, fasting plasma glucose, triglycerides and blood pressure were greater in subjects with MS as expected.

Plasma adiponectin had a similar pattern as observed in subjects from the Porto Alegre cohort. It was lower in the presence of MS (8.2 [5.5 – 13.8 μg/mL] vs 12.8 [9.0 – 20.5 μg/mL]; median [P25-P75], p = 0.005) (Figure 1A) and it significantly decreased with an increasing number of MS criteria (Figure 1B). Furthermore, when participants were stratified into three groups by adiponectin tertiles, the prevalence of MS decreased from the lowest to the highest adiponectin tertile (tertile 1 = 96.4%, tertile 2 = 91.5%, and tertile 3 = 90.8%; P for trend = 0.001).

Comparing according to the presence or absence of each MS criterion, adiponectin levels were significantly lower in the presence of following criteria: high waist circumference (10.4 [7.1 – 12.6 μg/mL] vs 18.5 [11.9 – 26.3 μg/mL]; P = 0.001), low HDL-cholesterol (10.6 [5.5 – 13.7 μg/mL] vs 14.4 [7.8 – 17.2 μg/mL]; P = 0.010), and elevated triglycerides (6.7 [7.6 – 9.6 μg/mL] vs 10.3 [11.7 – 14.0 μg/mL]; P < 0.001). However, this relationship was not found with elevated glucose (11 [5.6 – 14.0 μg/mL] vs 12.3 [5.8 – 15.7 μg/mL]; P = 0.110) and elevated blood pressure (11.0 [5.7 – 14.1 μg/mL] vs 10.9 [5.5 – 14.5 μg/mL]; P = 0.570).

While analyzing the relationship between adiponectin and the different MS components, adiponectin concentrations were positively correlated with HDL-cholesterol (r = 0.371; P < 0.001) and negatively correlated with waist circumference (r = -0.317; P < 0.001), fasting glucose (r = -0.118; P = 0.015), and triglycerides (r = -0.359; P < 0.001). Similar to the Porto Alegre cohort, there was no significant correlation between adiponectin concentrations with diastolic and systolic blood pressures (Figure 2).

HMW adiponectin had similar results as observed with total adiponectin. It was lower in the presence of MS (4.9 [6.2 – 7.3 μg/mL] vs 7.8 [7.9 – 13.5 μg/mL]; median [P25-P75], p < 0.001) and it significantly decreased with an increasing number of MS criteria (p < 0.001). Furthermore, when participants were stratified into three groups by adiponectin tertiles, the prevalence of MS decreased from the lowest to the highest adiponectin tertile (tertile 1 = 96.2%, tertile 2 = 91.5%, and tertile 3 = 83.8%; P for trend = 0.007).

Comparing according to the presence or absence of each MS criterion, HMW adiponectin levels were significantly lower in the presence of following criteria: low HDL-cholesterol (4.9 [6.3 – 7.4 μg/mL] vs 7.7 [7.3 – 11.5 μg/mL]; P = 0.005), elevated triglycerides (4.0 [4.6 – 5.9 μg/mL] vs 6.8 [7.7 – 9.4 μg/mL]; P < 0.001), and elevated glucose (5.0 [6.4 – 7.5 μg/mL] vs 7.0 [6.7 – 10.6 μg/mL]; P = 0.035). However, this relationship was not found with high waist circumference (5.0 [6.2 – 7.3 μg/mL] vs 5.9 [7.0 – 10.6 μg/mL]; P = 0.065), and elevated blood pressure (5.0 [6.5 – 7.7 μg/mL] vs 6.0 [5.8 – 9.3 μg/mL]; P = 0.170).

While analyzing the relationship between adiponectin and the different MS components, HMW adiponectin concentrations were positively correlated with HDL-cholesterol (r = 0.378; P < 0.001) and negatively correlated with waist circumference (r = -0.246; P < 0.001), fasting glucose (r = -0.128; P = 0.008), and triglycerides (r = -0.320; P < 0.001).

### Association between MS and adiponectin levels while adjusting for possible confounders

#### Porto Alegre cohort

To confirm the relationship between adiponectin levels and the presence of MS, we performed three different multiple Poisson regression models with MS as the dependent variable. In the first model an increment of 1-SD of adiponectin levels was significantly associated with a lower prevalence ratio of MS (0.84 [95% CI 0.75 – 0.93; P = 0.001]) while adjusting for sex and age. In the second model, when US-CRP and HOMA-IR were added to the model, 1-SD of adiponectin remained associated with a lower prevalence ratio of MS (0.88 [95% CI 0.79 – 0.97; P = 0.012]). The same is true while replacing HOMA-IR by ISI Matsuda in the model (0.88 [95% CI 0.80 – 0.97; P = 0.011]). Adding separately smoking status, alcohol consumption, physical activity, and waist circumference to this model did not change these results.

#### São Paulo cohort

We replicated similar models with data from the São Paulo cohort and observed similar results. In the first model, 1-SD of adiponectin levels was significantly associated with a lower prevalence ratio of MS (0.94 [95% CI 0.90 – 0.97; P = 0.001]) while adjusting for sex and age. In the second model, 1-SD of adiponectin remained associated with decreased prevalence ratio of MS (0.93 [95% CI 0.88 – 0.98; P = 0.001]) when HOMA-IR were added to the model. Adding separately smoking status, alcohol consumption, physical activity, and waist circumference to this model did not change these results.

## Discussion

The present study assessed the relationship between adiponectin levels and MS in a two-stage study using data from two cohorts. We demonstrated that lower circulating total and HMW adiponectin levels were associated with the presence of MS. Decreasing total and HMW adiponectin plasmatic levels were related with an increasing number of MS criteria in both cohorts. This association was independent of age and sex, smoking status, alcohol consumption, physical activity, waist circumference, insulin sensitivity, and subchronic inflammation as shown while adjusting for confounders in different multivariate models with data from the Porto Alegre cohort. In order to confirm these findings, we applied the same approach in an independent cohort of subjects with different clinical characteristics who were being investigated for coronary artery disease in São Paulo. The confirmation of the same associations in these two different cohorts would underscore that this was not a spurious finding. Additionally, total adiponectin levels were lower in the presence of each MS criterion in the Porto Alegre cohort whereas similar findings were observed in the São Paulo cohort, except for the elevated blood pressure and elevated glucose criteria. We found similar findings with HMW adiponectin levels which were lower in the presence of low HDL-cholesterol and elevated triglycerides criteria. Different from total adiponectin, HMW adiponectin levels were lower in the presence of elevated glucose criterion whereas their levels were not different in the presence of high waist circumference criterion. Since HMW adiponectin levels were inversely related with waist circumference, we believe that HMW adiponectin levels were not statistical lower in the presence of MS as a matter of sample size (P = 0.065).

Our results corroborate the findings of other studies which have analyzed the relationship between adiponectin levels and the MS [6,17]. Vega and Grundy showed that overweight and obese men with a high adiponectin/leptin ratio have a lower triglyceride, triglyceride/HDL levels and higher insulin sensitivity than those with a lower adiponectin/leptin ratio (6). Besides, Mente et al. suggested a possible causal relationship between low serum adiponectin levels and insulin resistance as measured by HOMA-IR (15).

As an extension to these studies, our findings suggest that adiponectin is inversely related to MS ratios while adjusting for possible confounders. We also tried to explore the possible factors related to lower adiponectin levels by the presence of MS. Lower adiponectin levels were observed with increasing US-CRP and HOMA-IR whereas higher adiponectin levels were related to increasing insulin sensitivity. Several studies have demonstrated that adiponectin is related to insulin sensitivity, since it sensitizes hepatocytes to the effects of insulin, suppressing hepatic glucose output [18]. Adiponectin also promotes fatty acid oxidation in the liver and adipocytes, decreasing triglycerides levels [19-22] and increasing glucose uptake by skeletal muscles [23,24].

Central obesity has been related to adipose cell enlargement and the development of a proinflammatory state [25]. We have found a significant and inverse correlation in both cohorts between waist circumference and adiponectin plasma levels. Additionally, low adiponectin levels were related to high US-CRP, a marker of subchronic inflammation. In fact, the hypoadiponectinemia, per se, may partly determine the proinflammatory state found in subjects with MS in our study, especially those with central obesity.

The strongest relationship between adiponectin levels and MS criteria was observed with HDL cholesterol. Some studies suggest that the association between adiponectin and HDL cholesterol may result from the effect of adiponectin in reducing triglycerides, apo A-I fractional catabolic rate [26,27], and hepatic lipase activity [28,29].

Although we found that decreasing adiponectin levels were related to increasing blood pressure in the Porto Alegre cohort, we were not able to find the same results in the São Paulo cohort with both, the total and HMW adiponectin. Additionally, we believe that in São Paulo, where subjects were investigated for coronary artery disease by coronary arteriography, a high rate of anti-hypertensive medications used by this population affected this relationship by their direct action on adiponectin levels and by their action on blood pressure.

There are potential limitations to our study. Firstly, the cross-sectional study design makes it difficult to infer causal relationship between low total and HMW adiponectin levels and MS. Secondly, the subjects are not representative of the general population, since they were referred for assessment and evaluation of MS in Porto Alegre and to coronary artery disease confirmation by coronary angiography in São Paulo. However, by using two samples with completely different profiles, we were able to replicate our results which strength the direction of our findings. Additionally, our study goes in the same direction of others that have shown an effect of adiponectin in regulating lipid and glucose metabolism, which corroborates our findings [6,17].

## Conclusions

In conclusion, total and HMW adiponectin levels not only are lower in the presence of MS, but it also decreases by increasing number of MS criteria. These levels are partly determined by their relationship with HDL cholesterol, triglycerides and abdominal adiposity. Furthermore, chronic inflammation and insulin resistance may contribute to the decrease in adiponectin levels. Longitudinal data of prospective population based studies might be used to understand the role of adiponectin in the development of MS.

## Abbreviations

MS: Metabolic syndrome; CVD: Cardiovascular disease; ABPM: Ambulatory 24-h blood pressure monitoring; OGTT: Oral glucose tolerance test; HbA1c: Glycated hemoglobin; us-CRP: Ultra-sensitive C-reactive protein; HOMA IR: Homeostatic model assessment of insulin resistance; Matsuda ISI: Matsuda insulin sensitivity index; NGT: Normal glucose tolerance; FPG: Fasting plasma glucose; IFG: Impaired fasting glucose.

## Competing interests

None of the authors had a conflict of interest.

## Authors’ contributions

1) Designed research: ADvonF, FG; 2) conducted research: ADvonF, AFR, FG; 3) analyzed data or performed statistical analysis: ADvon F, FG; 4) wrote paper: ADvonF, LEG, AFR, LHC, FG; 5) had primary responsibility for final content: ADvonF, FG; 6) critical review of the manuscript: all authors. All authors read and approved the final manuscript.
